# 

**Published:** 1867-12

**Authors:** 


### PENNSYLVANIA COLLEGE OF DENTAL SURGERY.

### THE ELEVENTH ANNUAL SESSION 1867–68.

### FACULTY.

J. D. WHITE, D.D.S., Emeritus Professor.
T. L. BUCKINGHAM, D.D.S., Prof. of Chemistry and Metallurgy.
E. WILDMAN, D.D.S., Prof. of Mechanical Dentistry.
G. T. BARKER, D.D.S., Prof. of Principles of Dental Surgery and Therapeutics.
W. S. FORBES, D D S., Prof. of Anatomy and Physiology.
JAMES TRUMAN, D.D.S., Prof. of Dental Physiology and Operative Dentistry.
EDWIN T. DARBY, D.D.S., Demonstrator of Operative Dentistry.
J. M. BARSTOW, D.D.S., Demonstrator of Mechanical Dentistry.

The regular Course will commence on the first Monday of November and continue until the first of March ensuing. During October the Laboratory will be open, and a Clinical Lecture delivered every Saturday, by one of the Professors, at 3 o'clock, P. M. The most ample facilities are furnished for a thorough course of practical instruction.

| | |
|---|---|
| Tickets for the Course, Demonstrators' Tickets included, | $100 |
| Matriculation Fee, - - - - - - - - | 5 |
| Diploma Fee, - - - - - - - - - - - | 30 |

For further information address

T. L. BUCKINGHAM, Dean,
243 North Ninth Street, Philadelphia.

### Patent Notice!

*The BUFFALO DENTAL MANUFACTURING COMPANY,* having purchased from ***E. A. L. ROBERTS,*** his entire interest in all of his Patents for

### Dental Vulcanizing Apparatus,

And the Patent issued to ***S. W. WARREN,*** which in connection with the Patents previously held by them, cover every form of the ***Single Chamber Vulcanizers*** now in use, together with all desirable features in regard to Thermometers, Heating Apparatus, Modes of Fastening, &c., &c.

***Now Therefore,*** the object of this is to notify Dentists, Dealers and Manufacturers that our legal rights will hereafter be strictly enforced.

The litigations thus far carried on have developed features which rendered this the only course for us to pursue.

Parties wishing to manufacture, use or sell must procure the proper license, and pay a reasonable share of the very great expense we have been compelled to incur.

Buffalo, October 19, 1867. Dec. '67.

### BALTIMORE COLLEGE OF DENTAL SURGERY

TWENTY-EIGHTH ANNUAL SESSION, 1867–68.

---

### FACULTY.

THOMAS E. BOND, A.M., M.D., Prof. of Pathology and Therapeutics.
PHILIP H. AUSTEN, A.M., M.D., D.D.S., Prof. of Dental Science and Mechanism.
FERDINAND J. S. GORGAS, A.M., M.D., D.D.S., Prof, of Dental Surgery
A. SNOWDEN PIGGOT, A.M., M.D., Prof. of Chemistry and Metallurgy.
RUSSELL MURDOCH, A.M., M.D., Prof. of Anatomy.
HENRY REGINALD NOEL, A. M., M. D., Professor of Physiology.
HUGH McGINNIS GRANT, D.D.S., Demonstrator of Operative Dentistry.
THOMAS SOLLERS WATERS, D.D.S., Demonstrator of Mechanical Dentistry.
CLAUDE BAXLEY, M. D., Demonstrator of Anatomy.

The *Twenty-Eighth* Annual Session will commence on the fifteenth of October, 1867, and close on the fifteenth of March, 1868.

Twenty-one Lectures are delivered every week throughout the session, besides the hours spent in actual practice.

| | |
|---|---|
| Lecture and Demonstration Fees, | $115 |
| Matriculation Fee (paid only once) | 5 |
| Diploma Fee. | 30 |

For information, address

F. J. S. GORGAS, M.D., Dean of the Faculty.
*No.* 43 *Hanover St., Baltimore, Md.*

---

### CHARLES ABBEY & SONS

MANUFACTURERS OF

### DENTISTS' FINE GOLD AND TIN FOIL,

***Nos. 228 and 230 Pear St.,***

PHILADELPHIA.

The attention of Dentists is invited to our FINE GOLD FOIL, which is prepared under our constant personal supervision. Our Nos. are 4, 5, 6, and 8.

We are also manufacturing an ADHESIVE FINE GOLD FOIL, Nos. 4 5 and 6.

ALL our Gold Foil is manufactured from ABSOLUTELY PURE GOLD prepared expressly for the purpose with great care, by ourselves.

### Dentist's Refined Tin Foil constantly on hand

CHARLES ABBEY,

WM. R. ABBEY,

CHAS. O. ABBEY

### NEW YORK
### COLLEGE OF DENTISTRY.

---

### FACULTY.

ELEAZAR PARMLY, M. D., F. C. D.,
Emeritus Professor of the Institutes of Dentistry.

WM. H. DWINELLE, M. D., D. D. S.,
Professor of Dental Histology.

EDWIN J. DUNNING,
Professor of Operative Dentistry.

NORMAN W. KINGSLEY,
Professor of Dental Art and Mechanism.

J. SMITH DODGE, Jr., M. D. D. D. S.,
Professor of Dental Pathology and Therapeutics.

FANEUIL D. WEISSE, M. D.
Professor of Descriptive and Comparative Anatomy. and of Oral Surgery.

RUFUS KING BROWNE, M. D.,
Professor of Experimental Physiology and Microscopic Anatomy.

P. H. VAN DER WEYDE, M. D.,
Professor of Chemistry and Metallurgy.

### ASSISTANTS.

GEO. ELIAS HAWES, M. D.,

Operative Dentistry.

ALEX. W. STEIN, M. D.,
Anatomy and Oral Surgery.

R. M. STREETER,

Mechanical Dentistry.

ADOLF OTT, Ph. D

Chemistry and Metallurgy.

### BOARD OF CLINICAL LECTURERS.

Dr. JOHN B. RICH, New York, *President.*

DR. JOHN ALLEN, New York

" W. B. ROBERTS, New York.

" EHRICK PARMLY, New York.

" W. W. SHEFFIELD, New Lond.

" WM. H. ALLEN, New York.

" D. H. GOODWILLIE, New York.

" BENJAMIN LORD, New York.

Dr. W. W. ALLPORT, Chicago.

" GEO. E. HAWES, New York.

" A. HILL, Norwalk, Conn.

" J. N. H. WALBRIDGE, N. Y.

" L. G. BARTLETT, New York.

" J. T. METCALF, New Haven.

" R. W. VARNEY, New York.

Dr. FRANK ABBOTT, N. Y., *Secretary.*

The plan of organization of this College provides for a course of instruction, didactic and demonstrative, by the above-named Faculty and their Assistants, in Practical Dentistry and collateral sciences; and also a Dental Infirmary, where clinical instruction will be given by the Board of Clinical Lecturers, which comprises a large number of the most skillful gentlemen in the profession. This Board will give daily Clinics during the entire session. The advantages to the student of such a combination in acquiring a thorough and practical knowledge of the science and art, it is believed, cannot be excelled. The session will open on Tuesday, October 15, 1867, and continue until the middle of March following.

Tickets for the course, $150, Matriculation, Demonstrators, and diploma fees included. For further information. see College Announcement, or address the Dean of the Faculty. NORMAN W. KINGSLEY, Dean,
*No. 25, West 27th St., New York.*

ELEAZAR PARMLY,

*Pres't Board of Trustees.* Aug. 4 mos.

### PHILADELPHIA DENTAL COLLEGE

108 North Tenth St., above Arch.

FIFTH ANNUAL SESSION, 1867 68.

---

### FACULTY

HENRY MORTON, A.M., Ph. D.

Emeritus Professor of Chemistry.

C. A. KINGSBURY, D.D.S.,
Professor of Dental Physiology and Operative Dentistry

D D. SMITH, D.D.S.,
Professor of Mechanical Dentistry and Metallurgy.

J. H. McQUILLEN, D.D.S.,
Professor of Institutes of Medicine.

J. FOSTER FLAGG, D.D.S.,
Professor of Institutes of Dentistry.

ALBERT R. LEEDS, A. M..
Professor of Chemistry.

JAMES E. GARRETSON, D.D.S.,
Professor of Principles and Practice of Surgery.

HARRISON ALLEN, M.D.,
Professor of Anatomy.

---

WM. C. HEAD, D.D.S.,
Demonstrator of Operative Dentistry

WM. P. HENRY, D.D.S.,
Demonstrator of Mechanical Dentistry.

The College will be opened on the first Monday of October and preliminary lectures delivered every day until the 15th of the month, when the regular course of lectures will commence and continue until the 15th of the ensuing March. In addition to the instruction from the various chairs, ample opportunity will be afforded for obtaining a practical knowledge of Surgery, and Operative and Mechanical Dentistry.

### FEES.

| | |
|---|---|
| Matriculation (paid but once) - - - - - - | $5 00 |
| Tickets for the *entire* Course, including the Demonstrators' | 100 00 |
| Diploma - - - - - - - - - - - | 30 00 |

For further particulars, address J. H. McQUILLEN, Dean,
Post Office Box, 2155, Philade p

